# Extra Virgin Olive Oil (EVOO) Components: Interaction with Pro-Inflammatory Cytokines Focusing on Cancer and Skeletal Muscle Biology

**DOI:** 10.3390/nu17142334

**Published:** 2025-07-16

**Authors:** Daniela De Stefanis, Paola Costelli

**Affiliations:** Department of Clinical and Biological Sciences, University of Turin, 10125 Turin, Italy; daniela.destefanis@unito.it

**Keywords:** EVOO, cancer, cytokines, cachexia, skeletal muscle

## Abstract

The advantages of extra virgin olive oil (EVOO) intake as part of a varied, healthy and balanced diet were demonstrated by many epidemiological studies. In particular, several components present in EVOO, such as tocopherols, carotenoids and phenolic compounds, play an important protective role in mitigating inflammatory diseases, atherosclerosis, neurodegenerative diseases and cancer. The protective effect exerted by EVOO was proposed to be accounted for by its antioxidant, anti-inflammatory or anti-proliferative properties. The present review will focus on the interactions among EVOO’s components and pro-inflammatory cytokines, aiming to reveal the mechanisms potentially involved in the anticancer action of EVOO. Cancer patients very frequently develop a devastating syndrome known as cachexia, which negatively impinges on their outcome. The main features of cachexia include progressive body weight loss, fat and muscle wasting, and dysmetabolism, all of which partially result from the onset of systemic inflammation. In this regard, the possibility that EVOO could be beneficial to cancer patients by mitigating cachexia will be reviewed, focusing on the skeletal muscle.

## 1. Introduction

Olive oil is one of the most ancient foods. Together with the olive tree, it has been known for thousands of years, and it was always used as a seasoning and cooking medium. In the last forty years, however, nutritional facts about the different types of olive oil were deeply investigated and EVOO is now considered one of the most relevant components of the Mediterranean diet.

The chemical composition of olive oils particularly depends on several factors linked to the specific cultivar, to peculiar environmental features as well as to both production and transformation processes, with the latter considered highly relevant in terms of the quality of the final product. EVOO is obtained from the fruit of the olive tree by mechanical crushing in the absence of solvents or chemical processes, followed by washing, decantation or centrifugation, and filtration [1]. EVOO is particularly enriched in healthy nutrients such as mono-unsaturated fatty acids, oleic acid in particular, essential fatty acids and bioactive compounds such as polyphenols, phytosterols, squalene, etc. While these latter compounds represent about 2% of EVOO’s composition, they are crucial to the definition of some organoleptic properties of EVOO, such as the taste and flavour. In addition, they are mainly responsible for the beneficial action of EVOO, being endowed with both antioxidant and anti-inflammatory activity. In this regard, epidemiologic data showed that regularly consuming EVOO was associated with protection against cardiovascular diseases, neurodegenerative and chronic inflammatory diseases and cancer.

## 2. Olive Oil Components

### 2.1. Biological Activity of EVOO

**Figure 1:**
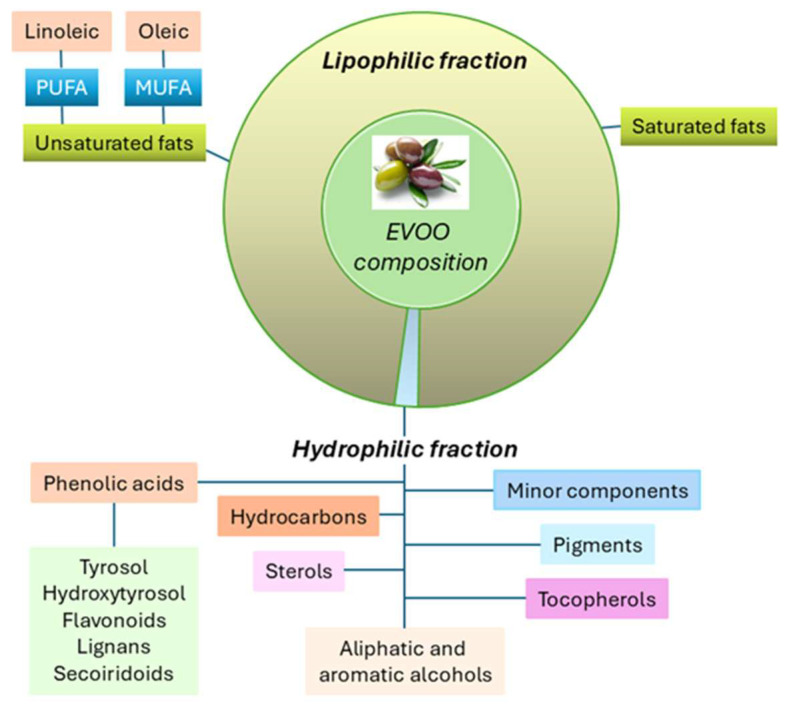
EVOO’s composition.

EVOO is mainly composed (98–99%) of a lipophilic fraction characterized by a high content of monounsaturated fatty acids (MUFAs), ranging from 65% to 83%, with oleic acid being the predominant MUFA. Additionally, EVOO contains polyunsaturated fatty acids (PUFAs), such as linoleic acid. The remaining 1–2% is represented by a hydrophilic fraction containing tocopherols, hydrocarbons, polyphenols, secoiridoids, aldehydes, ketones, beta-carotene, esters, alcohol, sterols, lignans, flavonoids, etc., also referred to as EVOO derivatives (Figure 1) [1].

Several studies demonstrated the preventive properties of EVOO against atherosclerosis, cardiometabolic disorders, neurodegenerative diseases, and cancers [2,3,4,5,6]. These beneficial properties are mainly linked to the polyphenols present in EVOO, such as tyrosol, hydroxytyrosol (HT), oleuropein (OLE), and oleocanthal (OC), which were shown to display anticancer activity and to counteract oxidative stress and inflammation (Table 1) [7,8,9].

Olive oil forms a micellar solution, allowing most phenolic compounds in EVOO to pass through the mouth and stomach largely unchanged. These compounds then reach the small intestine and colon [10], where they are directly absorbed, metabolized, and distributed to tissues, or transformed by the gut microbiota [11,12,13]. In the intestinal tract, hydroxytyrosol and tyrosol are assimilated at rates ranging from approximately 40% to 95%, depending on the dose [14]. While secoiridoids are highly stable in the mouth, they experience significant loss in the stomach, duodenum, and colon, with the recovery rates in the duodenum ranging between 7% and 34%. Their absorption involves processes such as glycosylation and the cleavage of glycosidic bonds. Additionally, some secoiridoids, like oleacein, are likely absorbed in the small intestine, primarily through passive diffusion across the intestinal cell membranes [10].

The 2011 PREDIMED study showed that EVOO’s consumption can improve glucose metabolism, preventing the onset of diabetes [15]. A subsequent PREDIMED study (2018) involving people at high risk of cardiovascular events showed that such a risk was lower in patients on a diet supplemented with EVOO than in those assigned to a low-fat diet [16]. A different study showed that metabolic markers, the abdominal fat distribution and the levels of pro-inflammatory cytokines in patients affected by metabolic syndrome were improved by the consumption of EVOO for 60 days [17]. Furthermore, in patients presenting with obesity and prediabetes, EVOO rich in OC and oleacein (OA) was more effective than a common olive oil in promoting body weight loss, improving fasting glucose and redox homeostasis, and decreasing circulating interferon-γ (IFN-γ) [18].

**Table 1:** Main bioactivities of EVOO’s polyphenols. Symbols: ↓ reduce; ↑ increment.

Activity Category	References	Polyphenols	Effects	Range of Concentration
Antioxidant	[2,4,5,7,8,9,19]	Hydroxytyrosol, Oleuropein, Tyrosol	Free radical scavenging ↓ oxidative stress in cells and tissues.	*In vitro* 10–100 μM *In vivo* 10–50 mg/kg/day
Anti-inflammatory	[3,4,5,6,7,19]	Oleuropein, Hydroxytyrosol, Oleocanthal,	↓ Cytokines (TNF-α, IL-6) ↓ NF-κB pathway ↓ inflammation markers	*In vitro* 10–100 μM
Antitumoral	[6,20,21]	Oleuropein, Hydroxytyrosol, Oleocanthal	↑ apoptosis ↓ proliferation of various cancer cell lines (e.g., colon, breast, hepatoma, melanoma) Modulates miRNA expression profiles	*In vitro* 20–100 μM. *In vivo* doses depend on model systems.
Gut Microbiota Modulation	[2,7,22,23]	Hydroxytyrosol, Oleuropein	Promotes beneficial bacteria growth (*Lactobacillus*, *Bifidobacterium*) improves gut health	Dietary supplementation (~50 mg/day)
Atherosclerosis and Cardiometabolic Disorders	[3,4,8,24,25,26,27,28]	Oleuropein, Hydroxytyrosol	Improves lipid profiles ↓ vascular inflammation ↓ foam cell formation ↓ blood pressure	*In vivo* 10–50 mg/kg/dye In human studies by Mediterranean diet
Neurodegenerative Disease	[4,5,9,29]	Hydroxytyrosol, Oleuropein	↓ neuroinflammation Potential to improve cognitive function	*In vitro* 10–50 μM *In vivo* doses vary depending on model

Due to their antioxidant properties, the polyphenols in EVOO reduced LDL oxidation and optimized the circulating cholesterol and triglyceride levels [30,31], counteracting the formation of atherosclerotic plaques [2]. Consistently, Casas et al. [24] demonstrated that adherence to the Mediterranean diet decreased the blood levels of inflammatory biomarkers related to the onset of atheromas in elderly people at high cardiovascular risk. Moreover, studies performed using a murine model of atherosclerosis showed that HT reduced both oxidative stress and inflammation in the vasculature, attenuating the progression of the disease [25]. The antioxidant and anti-inflammatory action of polyphenols and other compounds in EVOO also had beneficial implications for the blood pressure, thanks to a direct effect on the blood vessels (improved elasticity) [26], for the induction of nitric oxide (NO) synthesis by endothelial cells [32] and for the modulation of the expression of genes pertaining to the renin–angiotensin system [27].

Several studies showed the neuroprotective effect of EVOO’s components [29]. Experimental results obtained on a rat brain exposed to an ischemia–reperfusion protocol demonstrated that the antioxidant action of HT included the reduction of reactive nitrogen and oxygen (ROS) species, of lactate dehydrogenase levels and of pro-inflammatory cytokines [33,34]. Such effects were likely accounted for by the increasing hydrogen peroxide and hydroxyl radical scavenging, mainly through activation of the Nrf2 and JNK-p62/SQSTM1 pathways, and by inducing heme-oxygenase-1 [28,35,36,37]. Finally, in animals exposed to brain ischemia, HT improved both the blood flow and the connections among the brain regions, reduced inflammation and was able to positively impinge on the recovery of muscle function in the 15-day post-ischemia time window [38,39].

### 2.2. Epigenetic Effects of EVOO Derivatives

In the last few years, several pieces of evidence have demonstrated that the bioactivity of EVOO, and of the phenols it contains, can result from epigenetic-related mechanisms [40,41]. The latter may reflect DNA methylation, histone modification (acetylation and methylation), and noncoding RNAs such as microRNAs (miRNAs). Such epigenetic modifications are tissue-specific and stable enough to be inherited for a few generations [42].

The ability of EVOO to impinge on epigenetic regulation could contribute to preventing the development of some chronic diseases, such as cancer (see below) or cardiovascular events [40,41]. Consistently, patients on a Mediterranean diet showed changes in the methylation of the CpG sites of genes related to inflammation, diabetes, intermediary metabolism and a number of signal transduction pathways [43]. Similarly, studies performed using an experimental model of Alzheimer’s disease showed that HT was able to positively modulate the epigenetic dysregulation, resulting in an improvement of the cognitive impulsivity associated with the disease [44].

Many studies suggest that EVOO, or its derivatives, induces epigenetic changes by modulating miRNAs. In this regard, HT was reported to protect human chondrocytes from H_2_O_2_-induced cell death and the expression of some markers of osteoarthritis by reducing the miR-9 levels [45,46]. D’Amore et al. [47] compared the effect of high-polyphenol EVOO intake on the expression of the genes and miRNAs of peripheral blood mononuclear cells in healthy subjects and metabolic syndrome patients. In the former, but not in the latter, EVOO was able to downregulate the miRNAs involved in inflammation (miR-181b-5p and miR-23b-3p), insulin resistance (miR-107), and cancer (miR-19a-3p and miR-519b-3p), while resulted in the upregulation of miRNAs associated with anti-inflammatory (miR-23b-3p) and tumour-suppressing activity (miR-519b-3p). Finally, Carpi et al. [48] showed that modulations of NF-κB-related miRs, namely miR-34, miR-155 and let-7c, as induced by TNF-α in Simpson–Golabi–Behmel syndrome adipocytes, could be prevented by pretreatment with OC or OA.

## 3. EVOO’s Anticancer Activity

Tumour development is a multistep process that includes several events (neoplastic transformation, clonal expansion, angiogenesis and metastasis) that are strictly associated with inflammation [49,50]. Along this line, dietary EVOO was shown to exert a protective action against breast, colon and liver cancer.

Colorectal cancer (CRC) is a widespread tumour with a high mortality rate [51]. Several studies have confirmed an association between EVOO consumption and reduced onset of CRC [22]. A possible protective mechanism was proposed by Rodríguez-García et al. [52]. In their study, mice receiving three different types of high-fat diet (HFD) containing, as a unique fat source, coconut oil or sunflower oil or EVOO were compared. The EVOO-receiving mice showed a preventive effect against CRC development, which was associated with anti-inflammatory changes in the gut microbiota composition, such as decreased abundance of *Enterococcus* and *Pseudomonas* and an increased Firmicutes/Bacteroidetes ratio. By contrast, the coconut- and sunflower HFDs produced a dysbiosis that increased the CRC risk.

Consistently, the exposure to an EVOO extract enriched in OC and the ligstroside aglycone of hepatoma cells (HepG2, Huh7, Hep3B) reduced cell proliferation, increased cell death, and induced autophagy [20]. In addition, OC was shown to reduce liver tumour growth induced in nude mice by orthotopic implantation of human HCCLM3 cells, mainly by inhibiting STAT3 activity [53].

Garcia-Guasch et al. [54] showed that, compared to a diet rich in EVOO, a high-PUFA diet promoted the onset of breast tumour with a high degree of histopathological and proliferative alterations. By contrast, the high-EVOO diet, mainly through HT and/or OC, positively modulated the expression of proteins involved in different cell death pathways, favouring apoptosis. Similarly, OC was shown to suppress cell proliferation in two human melanoma cell lines, the highly tumorigenic A375 and the metastatic 501Mel, by downregulating Bcl-2 gene expression and reducing ERK and Akt phosphorylation [21].

Angiogenesis is an important factor for tumour survival, promoting both tumour growth and metastasis by providing nutrients and oxygen. The phenolic compounds from EVOO were demonstrated to reduce angiogenesis by modulating, in endothelial cells, the expression of proteins involved in proliferation, migration and invasion, adhesion, and survival [55,56]. Marrero et al. [57] demonstrated *in vitro* that OC and OA reduced the activity of matrix metalloproteinase (MMP)-2, thus inhibiting the ability of endothelial cells to invade matrigel and to form tubes, eventually resulting in endothelial cell death. More recently, treatment of HUVEC cells with an EVOO phenolic fraction was shown to reduce the expression levels of proteins associated with angiogenesis, inhibiting the ability of endothelial cells to migrate, adhere and form tubes. Such an effect was associated with reduced extracellular matrix degradation and enhanced endothelial cell apoptosis [58].

The chemopreventive role of EVOO in carcinogenesis could also be accounted for by modulations of the methylation status of genes involved in breast [59,60] and colon [61] cancer progression. Similarly, in rats hosting colon cancer, EVOO administration increased the methylation of the promoter region of the genes coding for NF-κB, VEGF, and MMP-9, downregulating their expression and counteracting tumour progression. Moreover, EVOO induced the hypomethylation of the promoter region of the genes encoding miR-143 and miR-145, upregulating their expression and eventually counteracting the NF-kB pathway. Finally, EVOO-induced hypomethylation was also observed in the promoter region of the genes coding for caspase-3 and caspase-9, increasing their expression and restoring apoptosis [62].

EVOO and its derivatives were shown to also inhibit breast cancer by modulating the expression of miRNAs, both *in vitro* and *in vivo*. In MDA-MB-231 and MCF-7 cells, OLE increased the expression of tumour suppressor miRNAs (miR-125b, miR-16, miR-34a) and pro-apoptotic genes (p53, p21 and TNFRS10B), while it decreased the expression of onco-miRNAs (miR-221, miR-29a, miR-21 and miR-155) and anti-apoptotic genes (bcl-2, mcl1) [63,64]. In addition, in MDA-MB-231 cells, OLE resulted in decreased levels of miR-194-5p and of its target PD-L1, one of the most relevant immune-escape-associated factors [65].

The antioxidant action of EVOO was shown to counteract the early stages of tumour development [7,8,9]; however, several studies reported that OLE, by inhibiting mitochondrial function, caused excessive ROS accumulation and apoptosis in different cancer cell lines (HepG2, A549, MCF-7, and MDA-MB-231) [66,67,68,69]. Similarly, the exposure of cancer cells to OC was reported to inhibit cell proliferation and colony formation, and to induce apoptosis, mainly by stimulating mitochondrial depolarization and intracellular ROS production [70].

Finally, EVOO-derived compounds appeared to also exert protective effects against chemotherapy-associated toxicity, one of the most relevant complications in the management of cancer patients. Indeed, both *in vitro* and *in vivo* experiments showed that secoiridoids such as OC, OLE, or tyrosol combined with chemotherapeutic drugs synergistically to reduce tumour cell proliferation [71,72,73,74,75], without affecting healthy cells [70,76,77].

### EVOO Interaction with Pro-Inflammatory Cytokines

**Figure 2:**
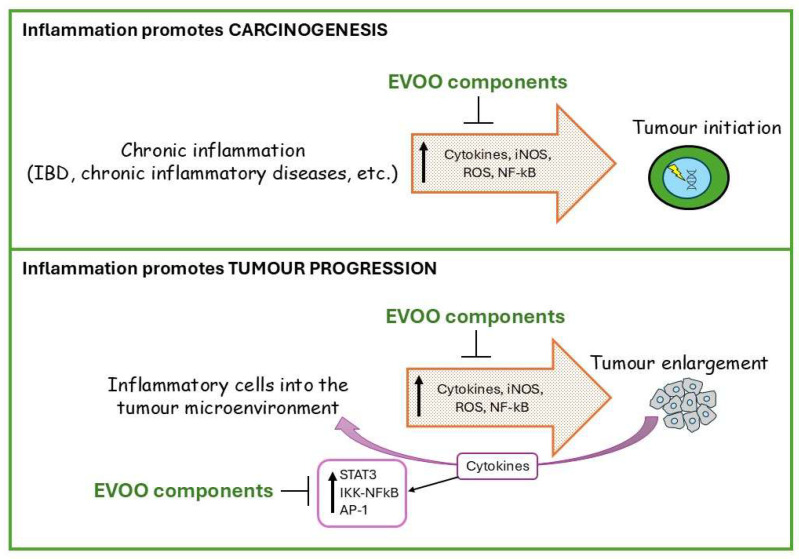
Modulation of carcinogenesis by the interaction of EVOO’s components with pro-inflammatory cytokines.

The onset and progression of most solid and hematopoietic tumours is associated with chronic inflammation that affects all stages of tumour growth (Figure 2) [49]. As an example, the low-grade inflammation occurring in patients with obesity and/or consuming an HFD supports gastrointestinal cancer development [78]. Similarly, pathogens, such as viruses and bacteria, frequently activate the inflammatory response, promoting the onset of cancer, as occurs in gastric cancer associated with *Helicobacter pylori* or in hepatocellular carcinoma favoured by hepatitis B/C viruses [79].

EVOO-derived polyphenols were reported to exert a protective effect against the onset of inflammatory bowel disease [80], a chronic disease of the gastrointestinal tract that can evolve into ulcerative colitis (UC) and Crohn’s disease (CD) [81]. Chicco et al. [82] analysed patients with IBD (84 with CD and 58 with UC) who were fed a Mediterranean diet for 6 months, showing that most of them experienced decreased liver steatosis and levels of inflammatory biomarkers (C-reactive protein and faecal calprotectin), improved quality of life and reduced disease activity. Moreover, daily consumption of EVOO decreased the inflammatory markers and improved the gastrointestinal symptoms in UC patients [83]. Finally, *in vitro* and *in vivo* experiments demonstrated that EVOO and its derivatives preserved the homeostasis of the intestinal epithelium by counteracting both oxidative stress and inflammation, maintaining or improving the gut microbiota and the immune response [23].

The exposure of differentiated colon cancer Caco-2 cells to oxysterols increased the production of pro-inflammatory cytokines and ROS; such an effect was inhibited by pre-treatment with a phenolic extract of EVOO, resulting in reduced IL-8, IL-6, and iNOS release in the culture medium [84]. Furthermore, in Caco-2 cells exposed to bacterial lipopolysaccharide (LPS), pretreatment with HT or tyrosol, or with their sulphate and glucuronide metabolites, inhibited iNOS expression and NF-κB activity [85].

In addition to favouring cancer onset, inflammation also plays crucial roles during cancer progression. Indeed, inflammatory cells infiltrate the tumour stroma and release mediators such as ROS, pro- and anti-inflammatory cytokines, chemokines, and growth factors. Moreover, cytokines can also be produced by the tumour itself, generating a tumour-promoting inflammatory loop that boosts its own development [49]. This whole scenario is regulated by multiple signalling pathways and transcription factors, with the dominant role being played by the axis impinging on IKK-NF-κB, JAK-signal transducer, STAT3, and MAPK-AP1, which can be influenced, directly or indirectly, by EVOO and its derivatives [86,87,88,89,90]. This was clearly shown by a study in which mice suffering from colitis induced by dextran sodium sulphate (DSS) were exposed to a standard diet, an EVOO-containing diet or an HT-enriched EVOO-containing diet. The EVOO-containing diet reduced by 50% the DSS-induced mortality observed in the standard diet group. Such an effect was associated with decreased levels of TNF-α and iNOS, reduced p38 MAPK activation and increased expression of the anti-inflammatory cytokine IL-10. This pattern was further improved in mice receiving the HT-enriched EVOO-containing diet [91].

Huguet-Casquero et al. [92] explored the effects of OLE alone or loaded on nanostructured lipid carriers in mice affected by acute colitis, showing that both treatments significantly decreased the myeloperoxidase activity, TNF-α, and IL-6 concentration in the colon mucosa, confirming the anti-inflammatory properties of EVOO derivatives, which likely reduce the risk of developing CRC. In the liver, inflammation and progressive tissue damage (e.g., steatosis, NASH, cirrhosis) result in preneoplastic lesions, which eventually evolve into hepatocellular carcinomas [93].

A number of studies showed that EVOO and its derivatives exert protective effects against liver damage, mainly by activating the Nrf2-dependent antioxidant response, counteracting inflammation through NF-κB inactivation, and inhibiting PERK, thus affording protection against endoplasmic reticulum stress, excess autophagy, and lipogenesis [19]. Several experiments showed that oleic acid and HT prevented lipid peroxidation in the rat liver [94], resulting in inhibition of hepatic fibrogenesis [95,96], liver steatosis [97,98,99], and hepatocyte ballooning [19]. Furthermore, HT downregulated the TNF-α and IL-6 mRNA and COX-2 expression in the liver of young male rats exposed to HFD, decreasing both steatosis and inflammation [19]. In addition, tyrosol administration to mice affected by NASH was shown to modulate the hepatic immune milieu, reducing inflammation and improving steatosis and fibrosis [100]. Finally, in HFD-fed mice, OLE lowered the LPS, TNF-α, and IFN-γ in the serum, and downregulated the intestinal and liver TLR4+ macrophages, reducing liver inflammation and steatosis [101]. Such effects were also proposed to be achieved by the OC-induced inhibition of the STAT3-dependent pathway, which was shown to result in reduced proliferation, epithelial–mesenchymal transition, migration and invasiveness and enhanced apoptosis in hepatocellular carcinoma cells as well as in an orthotopically implanted experimental HCC [53].

## 4. EVOO and the Skeletal Muscle

### 4.1. Skeletal Muscle Homeostasis

The skeletal muscle contains most of the body’s protein and represents about 40% of the human body weight [102]. Myogenesis occurs during embryonal, foetal and neonatal life. However, it can be reactivated during adult life in the case of muscle injury [103]. During muscle development, individual cells, the myoblasts, which derive from a population of multipotent mesodermal progenitors, fuse to form muscle fibres. The survival of these progenitor cells is guaranteed by the transcription factors Pax3 and Pax7, and their subsequent progression towards myoblasts is operationalized by the myogenic regulatory factors (MRFs: MyoD, Myf5, Mrf4, myogenin) [103,104]. During embryonic development, Myf5 is the first MRF to be expressed by the progenitor cells, which can subsequently differentiate into mononuclear myocytes that form the early muscle tissue of the embryo under the control of Mrf4 and myogenin. As the differentiation process progresses, stem cells expressing Pax3/Pax7, Myf5, and MyoD are recruited and contribute to the development of muscle tissue. At this point, Mrf4 is no longer expressed, while MyoD and myogenin drive the formation of primary myofibers through the fusion of myoblasts, in a process called primary myogenesis. Secondary myogenesis completes muscle formation: myogenin and Mrf4 drive the fusion of further proliferating myocytes into primary myofibers, which will then follow the specification into the oxidative (slow) or glycolytic (fast) phenotype [103,105]. In adults, myogenesis occurs following tissue damage through the recruitment of undifferentiated progenitor cells in a process that closely resembles the embryonal one [106].

The skeletal muscle performs several functions, such as force generation, movement, metabolic and endocrine regulation [103]. During fasting, the muscle can provide amino acids as precursors for gluconeogenesis, and its mass is directly related to bone density and mineralization [107]. Maintaining skeletal muscle mass, metabolism and function allows the body to cope with stressful conditions and helps prevent the onset of some chronic diseases [108].

Muscle mass depends on the balance between catabolic and anabolic processes, which are influenced by nutritional status, mechanical stress, age and concurrent pathologies. Muscle homeostasis is controlled and regulated through the pathways dependent on insulin/IGF1–Akt–mTOR (anabolic) and TGF-β/myostatin/BMP (catabolic), among others [109]. In particular, while protein catabolism is controlled by the Akt/FOXO pathway, protein synthesis is regulated by the Akt/mTOR axis. The interaction of these pathways is regulated, partially at least, by myostatin and insulin/IGF-1: overproduction of myostatin downregulates the Akt/FOXO axis, activating protein degradation and resulting in decreased muscle mass, while insulin/IGF-1 reduces muscle atrophy by switching off FOXO via Akt stimulation [110,111].

The complex interaction among Akt, FOXO and mTOR was demonstrated in mice characterized by skeletal-muscle-specific Akt deficiency, which showed a loss of muscle mass and function, associated with reduced oxidative metabolism. Such a pattern mainly resulted from decreased protein synthesis without significant changes in protein breakdown. In the same mouse model, mTORC1 activation or FOXO1 inhibition alone was not sufficient to rescue the loss of muscle mass, which is counteracted when both pathways are convergently (FOXO1 inhibited and mTORC1 activated) and simultaneously modulated [112].

As for the TGF-β/myostatin/BMP pathway, myostatin binding to its receptor activates ALK4/5/7, leading to phosphorylation of Smad2/3, which will interact with Smad4, forming a complex able to translocate into the nucleus. On the other side, when BMP ligands engage the receptor, ALK2/3/6 is recruited to activate Smad 1/5/8. The latter binds Smad4 as well, thus competing with Smad2/3 and counteracting the drift towards muscle atrophy [109].

### 4.2. Relevance of Pro-Inflammatory Cytokines

The pro-inflammatory cytokines can influence skeletal muscle homeostasis positively or negatively, depending on the level of cytokine expression, the type of producing cells and the type of signalling pathway(s) activated. Generally speaking, if cytokines are produced by the muscle itself in an autocrine manner, their levels are generally low and positively impinge on myogenic differentiation. If cytokines are released by infiltrating immune cells at skeletal muscle injury sites, muscle repair and regeneration are facilitated [113]. As an example, TNFα, the main mediator of the initial inflammatory response during skeletal muscle regeneration, was shown to promote C2C12 myoblast proliferation by modulating Myf5, and by upregulating myostatin mRNA, to reduce myoblast differentiation, suppressing premature differentiation after muscle injury [113]. Furthermore, TNF-α increased the expression of chemokines and cytokines, which influence immune cell recruitment, inflammation, and tissue regeneration [114].

IL-6 also, at low concentrations, exerts a pro-myogenic function. During load-induced compensatory hypertrophy, the IL-6 expression increased in both myogenic progenitors and myofibers [115,116], while the myogenesis-associated skeletal muscle hypertrophy was impaired in mice genetically lacking IL-6 [115]. Consistently, both IL-6 and the closely related cytokine LIF were expressed during skeletal muscle regeneration after acute injury [117,118,119,120]. Similarly to TNFα and IL-6, IFN-γ was reported to exert a positive function in regenerating muscles upon injury. Indeed, administration of an IFN-γ receptor-blocking antibody or deletion of IFN-γ in KO mice impaired muscle regeneration [121].

When cytokines are produced by activated immune cells or by damaged muscle, as in the case of dystrophy, their concentrations in the bloodstream are usually high, resulting in potent inhibition of myogenic differentiation. Along this line, the regenerative potential of satellite cells in dystrophic muscles was inhibited by elevated levels of TNF-α and by over-activation of NF-κB [122]. IL-6 is over-expressed in the muscle of patients affected by Duchenne muscular dystrophy and in mdx dystrophic mice. In the latter, treatment with antibodies against the IL-6 receptor was shown to enhance muscle regeneration, improving the dystrophic phenotype [123]. Furthermore, a reduction in the IL-6 levels was associated with attenuated muscle damage in adult mdx mice [124]. Inhibition or rescue of muscle regeneration was also reported in mice exposed to exogenous IFN-γ or to anti-IFN-γ antibodies, respectively [125]. Consistently, C2C12 myoblast proliferation and fusion were inhibited by exposure to an antibody against the IFN-γ receptor, thus favouring myogenic differentiation [121], which was inhibited by IFN-γ in both murine and human myoblasts cultures [126,127]. In the skeletal muscle, pro-inflammatory cytokines can modulate the balance between protein synthesis and degradation. Several pathologies, such as cancer, chronic heart failure, diabetes mellitus and sepsis, are associated with increased levels of pro-inflammatory cytokines, which could activate protein catabolism [128,129]. The imbalance towards protein degradation causes a loss of muscle mass, which a frequent occurrence of chronic diseases, including cancer. In this regard, cancer-induced muscle wasting is one of the most relevant features of cachexia, a complex syndrome that frequently complicates patient management.

### 4.3. Cancer Cachexia

Cancer cachexia is a multifactorial syndrome characterized by progressive body weight loss, reduction of muscle mass and adipose tissue, and metabolic alterations, which negatively impinges on patient prognosis, quality of life, tolerance to chemotherapy and survival [130,131]. In patients with cancer, cachexia develops differently, depending on the type of tumour [132,133,134]. Lung, liver and gastrointestinal tumours have a very high incidence of cachexia (between 70 and 90%) compared to leukaemia, breast cancer and favourable non-Hodgkin’s lymphoma (about 30%) [131,133]. The occurrence of cachexia in cancer patients increases with age, advanced Classification of Malignant Tumours (TNM) stage and sex, with males being more affected than females [133]. At present, cachexia is an unmet medical need and there are no effective treatments available. Along this line, investigations aimed at defining new suitable therapeutic approaches based on molecular evidences are warranted.

Skeletal muscle wasting featuring cancer cachexia was demonstrated to result from enhanced rates of protein degradation, mainly operationalized by the ubiquitin–proteasome and autophagic–lysosomal proteolytic systems, which is frequently associated with altered rates of protein [135]. The modulations of protein turnover were proposed to result, partially at least, from impaired energy metabolism. Indeed, the presence of a tumour, which competes with the host organism for energy and substrates to sustain its own metabolism, increases the patient’s energy expenditure [136,137]. In addition, it is now well accepted that cancer-induced muscle wasting is also characterized by altered mitochondrial function, resulting in reduced energy production and increased ROS levels [138].

An important contribution to the development of cachexia is made by pro-inflammatory cytokines such as TNF-α, IL-6, LIF and IFN-γ, produced by both the host and the tumour [139]. Indeed, cytokines are well known to promote muscle atrophy by NF-κB activation and by suppressing the Akt/mTOR pathway, resulting in enhanced protein breakdown, frequently associated with reduced protein synthesis rates [136]. Furthermore, cytokines act at the central level as well, promoting anorexia and the release of corticosteroids, which are well known to induce both protein and fat breakdown [136,140].

### 4.4. Impact of EVOO on Skeletal Muscle Homeostasis

Few *in vitro* and *in vivo* studies have analysed the mechanisms by which EVOO’s phenolic derivatives impinge on skeletal muscle homeostasis. For example, OLE counteracted the ROS increase caused in C2C12 myotubes by H_2_O_2_ and reduced the mitochondrial ROS generation in chicken muscle cells modulating the expression of Sirt1 and PGC1-α, with the latter being the master regulator of mitochondrial biogenesis [141,142]. HT administration to obese mice or to rats practising strenuous exercise was shown to improve the muscle mass and function by acting as an ROS scavenger, enhancing the activity of the endogenous antioxidant systems and stimulating mitochondrial biogenesis [143,144]. More specifically, the induction of mitochondrial biogenesis by EVOO was associated with enhanced activation of the signalling pathway dependent on AMP-activated protein kinase (AMPK), which triggers the overexpression of genes such as PGC1-α, NRF-1 and TFAM [145]. Along the same line, EVOO supplementation in animals exposed to a high-fat diet resulted in increased levels of the markers of autophagy, associated with a reduced pFOXO3/FOXO ratio [146]. Another study showed that both FOXO1 upregulation and the reduction of mTOR pathway activity were counteracted in diabetic pregnant rats consuming an EVOO-enriched diet [147].

Most studies have demonstrated the effectiveness of EVOO consumption in counteracting sarcopenia in the elderly. In particular, EVOO intake was proposed to activate anabolic pathways and to prevent mitochondrial damage and inflammatory processes responsible for sarcopenia [148,149].

Despite the observations reported above, there are still very few studies aimed at understanding if EVOO and its derivatives can counteract muscle atrophy. In this regard, experiments performed on C2C12 cells cultured in high glucose medium, which mimics the persistent hyperglucose environment of diabetes, showed that exposure to tyrosol protected myoblasts by reducing ROS production, resulting in increased cell proliferation, suppressed apoptosis, and restored ability to release angiogenic factors into the culture medium. Consistently, hindlimb ischemia in diabetic mice could be improved by recovering blood perfusion through tyrosol injection into the gastrocnemius muscle [150].

**Figure 3:**
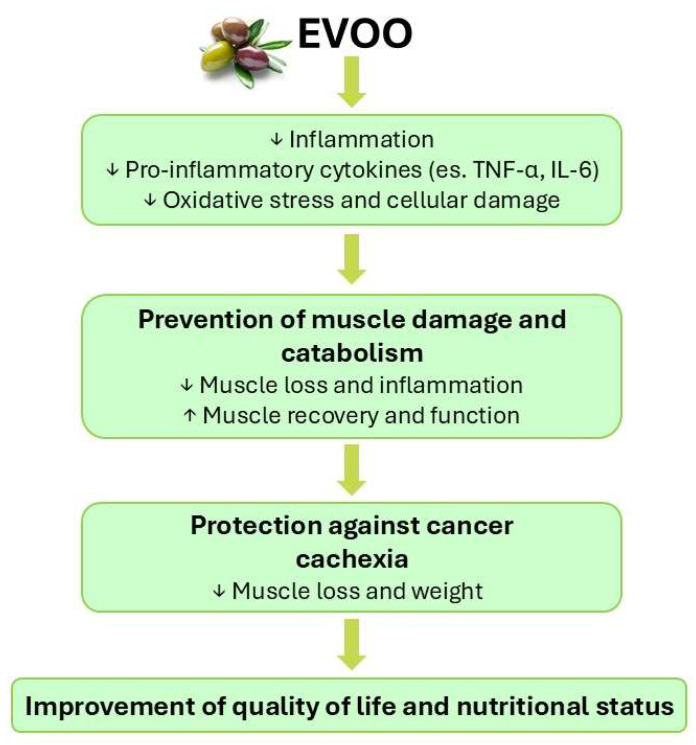
Potential role of EVOO in the prevention of cancer cachexia. Symbols: ↓ reduce; ↑ increment.

Few pieces of evidence are actually available concerning the possibility that the EVOO-enriched Mediterranean diet or EVOO components, known to exert a protective action against cancer both *in vitro* and *in vivo*, could also positively impinge on cancer-induced skeletal muscle wasting (Figure 3). Until now, only three clinical studies have analysed the correlation between the Mediterranean diet and cancer cachexia. In a study conducted in prostate cancer patients exposed to androgen deprivation therapy, in which obesity was a treatment-induced negative side effect, Baguley et al. [151] showed that those adhering to the Mediterranean diet experienced improvements in their quality of life and fatigue, as well as a reduction in their total body mass, fat mass and IL-8 levels, compared to patients on the standard diet.

Another study analysed the effects of a Mediterranean diet in patients affected by lung cancer, showing that it was able to reduce both the inflammation index and the C-reactive protein blood concentrations [152]. Finally, Bagheri et al. [153] compared two groups of CRC patients with cachexia, one given the Mediterranean diet and one fed their normal diet (controls). The loss of body weight, adipose tissue, lean body mass and muscle function were significantly improved in patients fed the Mediterranean diet compared to the control group. The circulating levels of TNF-α, C-reactive protein, and IL-6 were considerably reduced in the Mediterranean diet group, with an improvement in the general health status and exercise performance [153]. Recently, OC was reported to protect C2C12 myotubes against the reduction in size deriving from exposure to TNF-α or to the medium conditioned by cultured C26 cells (CM-C26), a tumour well known to induce cachexia in the host mice. Indeed, when added to TNF-α- or CM-C26-treated C2C12 cultures, OC restored the myotube morphology and size, also normalizing the expression of the accepted markers of protein degradation atrogin-1 and MuRF1. Furthermore, OC positively modulated the expression of Pax7, myogenin, and MyHC, three molecules involved in myogenesis [154]. Consistently, in a previous study, tyrosol was shown to counteract the negative effects exerted by dexamethasone in C2C12 myotubes. Indeed, morpho-functional analyses revealed that dexamethasone reduced the myotube size and induced an immature syncytia phenotype compared to the control cultures. Furthermore, the presence of dysfunctional mitochondria and the accumulation of autophagic vacuoles contributed to myotube degeneration and death. Administering tyrosol prior to glucocorticoid treatment mitigated the myotube damage and restored both mitochondrial and lysosomal function [155].

## 5. Conclusions and Future Perspectives

Numerous epidemiological studies have highlighted the health benefits of EVOO. Due to its antioxidant and anti-inflammatory properties, EVOO plays a crucial role in protecting against inflammatory diseases, atherosclerosis, neurodegenerative disorders, and cancer. In the present review, the potential interactions among EVOO, pro-inflammatory cytokines, cancer, and cachexia are highlighted. In this context, EVOO can help in reducing inflammation and oxidative stress, thereby acting on one side on cancer onset and progression, while on the other side, it could protect the skeletal muscle tissue, preserving its mass and function.

However, most of the available data come from *in vitro* reports, with very few studies performed on preclinical models or human beings. This is particularly relevant when taking into account issues such as EVOO’s polyphenol concentration, absorption, bioavailability and metabolism. In this regard, it must be considered that many of the bioactivities exerted *in vitro* by EVOO derivatives are not confirmed by *in vivo* experimental and clinical studies. Several factors could account for such a discrepancy: (i) the concentrations used *in vitro* are generally higher than those occurring in the human diet; and (ii) the amount of the bioactive compound that can reach the circulation can be markedly lower than expected. As an example, the concentration of non-metabolized hydroxytyrosol reaching the bloodstream is less than 1% of the amount introduced with the diet [156]. On the other side, the concentrations of metabolites derived from hydroxytyrosol and tyrosol can become markedly high, and they can share the biological effects with the original compounds, being able to modulate the intracellular signalling pathways that account for the effects of EVOO derivatives [157]. Just as another example, a high polyphenol dosage could result in pro-oxidant rather than antioxidant action, leading to altered mitochondrial function and enhanced mutation rates [158]. Finally, gender differences in terms of metabolism, the metabolic response to EVOO derivatives and the extent of the biological effects, as well as polymorphisms in the drug-metabolizing systems, are additional issues to be considered when approaching the use of EVOO derivatives *in vivo*. An additional note is that the effects of chronic assumption of EVOO polyphenols should be investigated.

Keeping in mind the potential limitations above, the existing knowledge suggests that EVOO might be a nutraceutical intervention useful for contributing to cancer prevention and to the management of the associated cachexia. Indeed, by safeguarding muscle, EVOO may lower the risk of cancer cachexia and assist patients in better tolerating anti-tumour therapies, ultimately enhancing their quality of life. Such a hypothesis is supported by studies showing that the combination of supplements such as essential amino acids, antioxidants, or other agents that promote muscle protein synthesis and reduce inflammation may be more effective than conventional treatments (chemotherapy and dietary interventions). For example, an association treatment for cancer cachexia, which included a polyphenol-enriched diet, antioxidants, medroxyprogesterone acetate, and celecoxib, was shown to be effective and safe in an early phase II study [159].

In recent decades, nanotechnology has become increasingly vital in improving the solubility, bioavailability, permeability, and stability of natural compounds and extracts [160]. Polymeric micelles have been developed to improve the intestinal permeability of OLE [161]. Encapsulating in polymeric micelles both hydrophobic and hydrophilic natural compounds prolongs the circulation time in the bloodstream and protects against chemical and enzymatic breakdown in the gastrointestinal tract, ultimately increasing their ability to cross the intestinal barrier, and reducing oral doses and potential toxicity.

On the whole, despite the still unclear issues above, the integration of EVOO into the diet, or the fortification of foods with EVOO polyphenols, could reveal a useful strategy for improving the management of cancer patients. Along this line, both preclinical and clinical studies are warranted.
